# Quantification and Degradation of 2,2-Dibromo-3-Nitrilopropionamide (DBNPA) in Bioethanol Fermentation Coproducts

**DOI:** 10.1007/s11274-022-03253-0

**Published:** 2022-03-29

**Authors:** J. V. Simpson, C. L. Wiatr

**Affiliations:** 1 National Corn-to-Ethanol Research Center (NCERC), Southern Illinois University-Edwardsville, Edwardsville, IL United States; 2 Chemir Analytical Lab, Pfizer, St. Louis, MO United States; 3 Buckman Laboratories, Memphis, TN United States

**Keywords:** Antibiotics, Bioethanol, Biocide, Bioluminescence, 2,2-dibromo-3-nitrilopropionamide, DBNPA

## Abstract

2,2-Dibromo-3-nitrilopropionamide (DBNPA) has been used as a biocide in industrial water applications due to its instantaneous antimicrobial activity and rapid chemical breakdown. In this study, DBNPA is considered a potential alternative for antibiotics used for bacterial control during corn-to-ethanol fermentation. A method using LC/MS/MS was developed to accurately quantify DBNPA in water. When this method was applied to quantify DBNPA concentration in a fermentation matrix, DBNPA was found to be unstable and to decay rapidly, preventing validation of the method or quantitation. This method was then used to evaluate the degradation rate of DBNPA in whole stillage, which is the nonvolatile residue produced by removal of ethanol from corn-based fermentation beer by distillation through the relative decrease in measured signal. In addition, a method was developed and validated to quantify bromide, one of the degradation products of DBNPA, in whole stillage using LC/MS/MS. The degradation rate of DBNPA in whole stillage was found to display first-order kinetics with a calculated half-life of 85 min. Laboratory analytical chemistry results on DBNPA degradation were confirmed based on a bacterial viability assay in field trials.

**Supplementary Information:**

The online version contains supplementary material available at 10.1007/s11274-022-03253-0

## Introduction

Fuel-ethanol production in the U.S. has grown from less than 2 billion gallons per year in 2000 to 15.8 billion gallons in 2019 [10]. A major byproduct of ethanol production from corn is distiller’s dried grains with solubles (DDGS). DDGS is used primarily as animal feed and consists of the nonfermentable components of the corn kernel (e.g., protein, oil, and fiber), unconverted starch, and nonvolatile fermentation products (e.g., yeast biomass, glycerol) [24]. During corn fermentation, bacterial contamination and spoilage of products are common problems since chemical and biological processing are not performed under aseptic conditions [26]. To prevent and treat bacterial infections, antibiotics, such as virginiamycin and penicillin, are often added to the fermentations [2,16, 26]. Recently, the FDA has become more concerned about antibiotic residues in DDGS, [12a, 12b] and as a result, interest in alternative strategies for controlling bacterial infections has increased in the ethanol industry. One such strategy is to apply a biocide such as 2,2-Dibromo-3-nitrilopropionamide (DBNPA) [29, 30, 31]. This biocide is an effective bactericide but does not affect the yeast employed to ferment corn to ethanol [29, 30, 31].

DBNPA is a brominated acetamide [15] originally applied as a seed fungicide [22]. More recently, DBNPA was used effectively to limit bacterial growth in different water applications, such as cooling water and paper processing [5, 17, 25]. In these applications, favorable characteristics of DBNPA included instantaneous antimicrobial activity and rapid chemical breakdown into relatively nontoxic byproducts [11, 29]. These characteristics would also be beneficial in the ethanol industry, [29,30, 31, 32] but the behavior of DBNPA has not been studied in this application. In particular, DBNPA decomposition in water treatment applications is dominated by two reaction pathways: pH-dependent hydrolysis and light-catalyzed reactions with reducing nucleophiles [11]. However, the relevance of these pathways in ethanol processes and operations is not known.

Whole stillage is comprised of nonvolatile residues produced by removal of ethanol from corn-based fermentation beer by distillation. It typically contains 95% water and 5% residual material from corn fermentation. This includes fermentation byproducts, residual fermentable sugars, and nonfermentable components of corn, such as protein, triglycerides and free fatty acids, and corn fiber. Because whole stillage is a critical intermediate product of the operations that eventually result in the production of DDGS, it was selected as the sample matrix for this study. Previous methods have utilized HPLC with UV detection to quantitate DBNPA in wastewater [11], but interference from other components of whole stillage precluded the use of this method in this matrix. Similar interference issues were encountered when conductivity detection was used to attempt to quantify bromide, a major decomposition product of DBNPA, in the whole stillage. To achieve the analytical goal of this study to measure DBNPA degradation in fermentation medium, we developed and validated quantification methods for DBNPA and bromide using liquid chromatography/mass spectrometry/mass spectrometry (LC/MS/MS). Subsequently, we introduced DBNPA to the fermentation process in field trials and measured the presence or absence of this biocide downstream in the corn-to-ethanol process using bioluminescence.

## Materials and methods

**Chemical Standards and Reagents** DBNPA was purchased from Sigma Aldrich (St. Louis, MO, USA; CAS# 10222-01-2) at 96% purity. Field trials used 20% active ingredient dissolved in propylene glycol manufactured by DOW Chemical Co. (Midland, MI.) LC/MS grade methanol, formic acid, ammonium acetate, sodium bromide, and N,N-1,4-diethyl-*p*-phenylenediamine (DPD) were also purchased from Sigma Aldrich. Purified (DI) water, with a measured resistance of 18.2 MΩ, was produced using a Millipore Synergy 185 (Millipore, Billerica, MA, USA). High-purity nitrogen, which was used as the nebulizing, sheath, and collision gas, was provided by a nitrogen generator (Peak Scientific, Billerica, MA, USA).

Frozen microorganism cultures of the bioluminescent bacterium *Aliivibrio fischeri* were obtained from Microtox (Azur Environmental, Carlsbad, CA). These were thawed in a water bath at 15.0 ± 0.5 °C, hydrated, adjusted osmotically to 2% NaCl with 22% NaCl in ultrapure water, mixed aseptically, and utilized immediately for tests on a Microtox 500 Analyzer, according to the instructions of the manufacturer [21] The photometric reading of the light production by *A. fischeri* was measured in a Microtox 500 (λ = 490 nm) and immediately recorded. The light output radiated by this bacterium is known as an indicator that is directly proportional to bacterial cell metabolic activity [21].

**Whole stillage supernatant** For laboratory studies, a whole stillage sample was selected from archived samples that were generated during a pilot plant trial that used typical conditions for the production of fuel ethanol and DDGS from corn. The pilot plant trial was performed at NCERC, and the sample was stored frozen at -20 °C until it was used in this study. Whole stillage supernatant was prepared by centrifuging approximately 50 ml of whole stillage at 22 °C at 3000 rpm for 5 min. The supernatant was filtered through a 0.45 μm syringe filter to remove colloidal suspended solids. The pH of the whole stillage sample was 4.6. Field samples of whole stillage were handled similarly.

**DBNPA Identification and Quantitation** DBNPA was analyzed in a methanol/water matrix with a Shimadzu SPD 20 HPLC system (Shimadzu Corporation, Kyoto, Japan) consisting of an autosampler, a degasser, two dual head pumps, and a thermostated column oven set at 40ºC connected to a triple quadrupole mass spectrometer (3200 QTrap, AB Sciex) equipped with an ESI turbo ion source. A binary solvent system was used: solvent A was 0.1% (w/v) formic acid in water, and solvent B was 0.1% (w/v) formic acid in methanol. An Insertsil ODS-4 C18 column (6 mm x 250 mm, 5 μm; GL sciences, Torrance, CA, USA) and a guard column (7.1 mm x 2.1 mm) were used for chromatographic separation. Chromatographic separation was performed at a flow rate of 0.9 mL min^− 1^ using a gradient elution program (T = 0 min, A: 95% B: 5%; T = 10 min, A: 75% B: 25%; T = 10–17 min A: 75% B: 25%; T = 17–21 min A: 60% B: 40%). The mass spectrometer was operated in negative mode under software control (*Analyst*, version 1.5, AB Sciex). DBNPA was detected using 238.8, 240.8, and 242.8 m/z ions, corresponding to the [M-H]^−^ ion of DBNPA with various bromide isotopes.

The method was validated by evaluating linearity, accuracy, and precision. Five external calibration standards were prepared through dilutions of a 200 ppm DBNPA stock solution prepared by dissolving an appropriate amount of DBNPA in a 50/50 methanol/water mixture using a volumetric flask producing the following concentrations: 25 ppm, 20 ppm, 10 ppm, 5 ppm, and 2 ppm. The correlation coefficient, r^2^ (or coefficient of determination), between the detector response and the concentrations of the external standards was 0.998. Due to the rapid degradation of DBNPA in whole stillage, the method could not be validated in this medium. Instead, the method was validated using a water/methanol matrix, in which DBNPA is relatively stable. The accuracy of the method was determined from the recovery of DBNPA in a series of spiked samples. Aliquots were made from the 200 ppm DBNPA stock solution in the following amounts: 500 µl, 100 µl, 50 µl, and 25 µl. The aliquots were then diluted with 50/50 methanol/water create spiked samples at the following concentrations: 100 ppm, 20 ppm, 10 ppm, and 5 ppm. In the case of spiked samples that were spiked with a concentration above the calibration range, the sample was diluted by a factor of 10 before submission for analysis. (See Table 1.)

**Table 1:** LC/MS/MS results from aliquots made from 200 ppm stock DBNPA solution and then diluted in 50/50 methanol/water. (See Materials and Methods.) Validation results of the DBNPA method for both accuracy and precision. Accuracy results are reported as a percent of the spike value detected in the spiked sample. Precision results are reported as the relative standard deviation of 5 injections from the same sample.

Accuracy		Precision		
**Sample ID**	**Recovery (%)**		***n***	**RSD (%)**
100 ppm	93.2	5 ppm	5	2.56
20 ppm	103.4	60 ppm	5	0.89
5 ppm	104.2			
2 ppm	98.3			

**Bromide Identification and Quantitation** Bromide concentration in whole stillage was determined with the same chromatograph and detection instruments as were used for DBNPA. Before instrumental analysis, the whole stillage sample was cleaned using a solid phase extraction procedure. The SPE cartridge (C18, Agilent, Santa Clara, CA, USA) was conditioned with methanol, then 50/50 methanol/water, the whole stillage sample was filtered through the cartridge, and the eluent was collected for direct injection on LC/MS/MS. A Dionex RFIC Ionpac AS22 (4 mm x 250 mm) ion chromatography column was used with 0.2% (w/v) ammonium acetate in water solution as the mobile phase. Identification and detection of analytes was performed by a triple quadrupole mass spectrometer (3200 QTrap LC/MS/MS, AB Sciex) equipped with an APCI ion source operated in negative mode with a mass transition of 79/79.

The method was validated by evaluating its linearity, accuracy, and precision. Five external calibration standards were prepared at concentrations of 12.4, 5.0, 2.5, 1.2, and 0.5 ppm bromide ions. The correlation coefficient (or coefficient of determination) of the external calibration curve was 0.999. Spiked samples were prepared to test the accuracy of the method by spiking an aliquot of concentrated sodium bromide into whole stillage supernatant. Spikes were prepared at the following concentrations: 6.8, 4.3, and 1.8 ppm bromide ions. Recovery for each spiked whole stillage sample was determined. Instrument repeatability was evaluated based on five injections of the 1.2 ppm standard and 4.2 ppm spiked sample. Relative standard deviations are shown in Table 2 for both sample types.

**Table 2:** Validation results of the bromide method for both accuracy and precision. Accuracy results are reported as a percent of the spike value detected in the spiked sample. Precision results are reported as the relative standard deviation of 5 injections from the same sample. See Methods.

Accuracy		Precision		
**Sample ID**	**Recovery (%)**		***n***	**RSD (%)**
6.8 ppm	104.4	1.2 ppm	5	2.144
4.3 ppm	108.3	4.3 ppm	5	3.905
1.8 ppm	99.1			

**DBNPA Degradation Time Trials** A whole stillage supernatant sample was spiked with DBNPA to make a 40 ppm DBNPA sample for the degradation study. The sample was kept at 15 °C in the dark within the autosampler between injections due to the photosensitivity of DBNPA. The sample was analyzed using the LC/MS/MS method we developed and validated for DBNPA in methanol/water.

A duplicate sample of whole stillage supernatant was spiked with DBNPA to an initial concentration of 40 ppm DBNPA for the degradation study. Instead of determining the DBNPA concentration, the bromide concentration was monitored. The method was modified to remove the SPE pretreatment phase due to the time requirements of the experiment.

**Field trials** Field sites in four states were surveyed to run proprietary trials. Ethanol plants were sought specifically where personnel had experience working with biocide techniques (as opposed to non-biocide, clean-in-place technologies) but also where personnel were not bound by contract or were not given exclusivity rights to test uncharacterized chemicals or biochemicals from another company or agency. Two corn-to-ethanol plants agreed to carry out a confidential trial. Before proceeding, they cleaned their equipment with permeate water from their reverse osmosis system. The absence of chlorine in the water was determined by using standard DPD testing in a HACH DR5000™ spectrophotometer. This was done to assure that chlorine was not present in the water to act as a biocide, and so it would not oxidize Br^−^ to HOBr and throw off our measurement of [Br^−^].

DBNPA was introduced into the fermenters as 200 ppm small quantity dosages, as product (20% active).[29] The temperature of the first fermenter was 32–33 °C and pH 4.6 ± 0.1 for 42 h; the second plant maintained its fermentation at 35 °C and pH 4.5 ± 0.1 for 46 h. In both fermentation trials, field samples were drawn by experienced plant personnel and dispensed into opaque, covered containers to avoid photodegradation of DBNPA. Samples were centrifuged, followed by filtration through 0.45 μm syringe filters as described above. The presence of DBNPA was determined by its effect on the bioluminescence radiated by *A. fischeri* culture, as measured in the Microtox 500 Analyzer, [21] and the results from the trial samples were compared with the control samples. The goal was to have data points from the field samples within 3–5% of each other.

## Results

**Degradation of DBNPA** was determined using LC/MS/MS. Table 1 lists the recovery for each spiked sample. Instrument repeatability was determined from five injections out of a standard (5 ppm) and spiked sample (60 ppm). For each, the relative standard deviation based on peak area was calculated for the set of runs. The limit of quantitation was not determined for this method.

Table 2 lists the recovery for each spiked whole stillage sample. Instrument repeatability was determined from five injections of the 1.2 ppm standard and 4.2 ppm spiked sample. Relative standard deviations are shown in Table 2 for both sample types.

Both developed analytical methods displayed a high degree of accuracy and reproducibility during validation. For both methods, recovery of the spikes at all levels was within 90–110% recovery of the spike. The RSD for both methods at both high and low analyte concentrations was below 5%. The results of the validation are shown in Tables 1 and 2 for DBNPA and bromide, respectively.

**Fig. 1:**
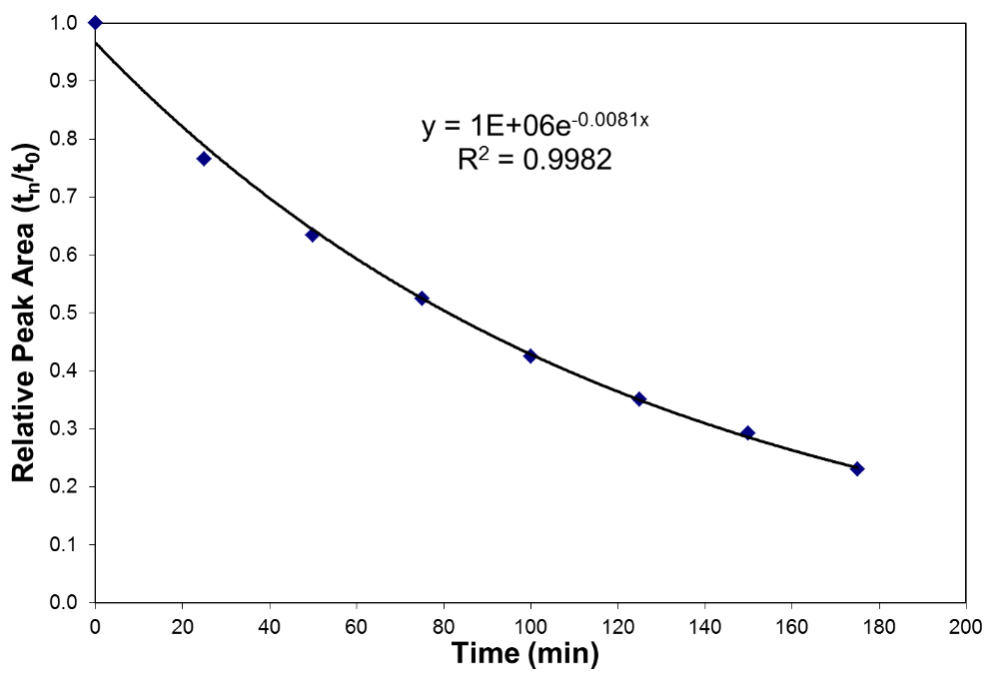
Decay of DBNPA in whole stillage. Each data point is a replicate injection from the same spike whole stillage sample.

**Fig. 2:**
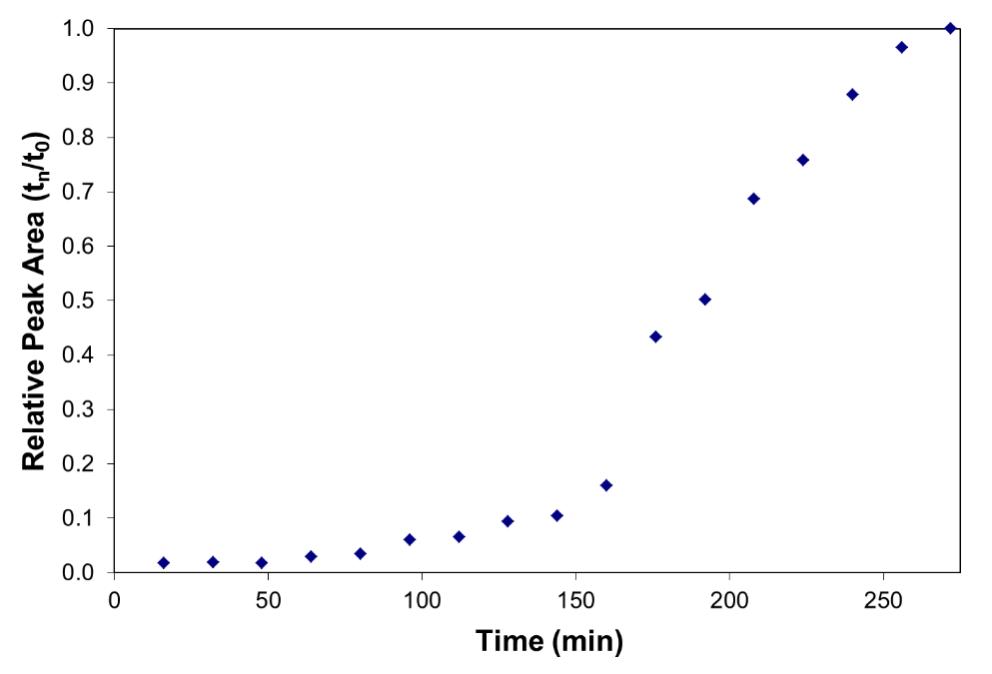
Change in the concentration of bromide in whole stillage detected as a function of time. Each data point is a replicate injection from the same whole stillage sample spiked with DBNPA.

Figure 1 shows the change in relative DBNPA signal area as a function of time in the whole stillage. The relative signal area of DBNPA decreased to approximately 20% of its initial value when incubated for 3 h at 15 °C in the dark, and the rate appeared to be first order in DBNPA concentration. The best-fit degradation rate coefficient (0.0081 min^− 1^) corresponds to a half-life of approximately 85 min. The results in Fig. 2 indicate that even though DBNPA degradation started immediately, bromide was only produced after a delay of approximately 150 min. After this relatively long lag period, bromide was rapidly released as a free ion.

**Fig. 3:**
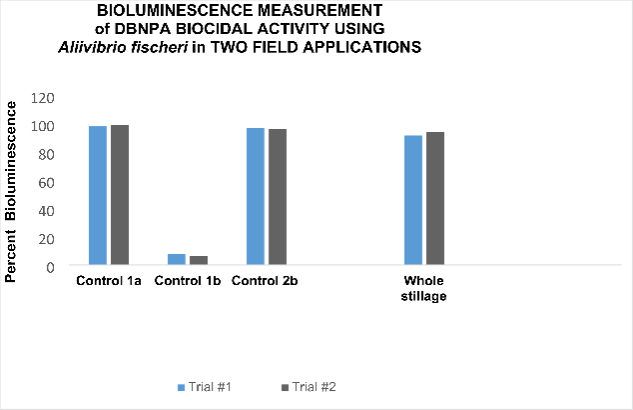
2,2-dibromo-3-nitrilo propionamide (DBNPA) was dosed as 200 ppmv of Bronam 20 in laboratory samples and plant fermentation media. Bioluminescent bacteria, *Aliivibrio fisheri*, were tested at 0 min to obtain a negative control reading (Control 1a) under ambient conditions. Samples that were exposed to DBNPA for 60 min in the in the laboratory yielded a decrease in bioluminescence (Control 1b) whereas readings of control samples remained the same. Similarly, after the fermentation process was completed in the plant, whole stillage was harvested, and tested. The results of DBNPA degradation in whole stillage were compared with the controls. Bar 2b represents a check of viability of fresh laboratory cultures prior to testing the whole stillage samples.

**Field trial results** In field trials, DBNPA was introduced at 200 ppm in each fermenter. After completion of fermentation, samples were drawn from whole stillage and prepared for analysis using the Microtox 500 instrument. (See Methods.) Bioluminescence measurements were used to determine the presence of DBNPA in the stillage and compared with laboratory controls. Exposure to a microbicide such as DBNPA causes a change (decrease) in luminescence, which is a byproduct of cellular respiration of photoluminescent bacteria. Changes in respiration directly relate to the toxicity of the biocide and to the inhibition of *A. fischeri* bioluminescence [21]. The results of field trials carried out at two corn-to-ethanol plants are provided in Fig. 3. Laboratory samples with and without DBNPA under ambient conditions were tested at 60 min, and 0 and 60 min, respectively. The results clearly indicate that the bioluminescence radiated by untreated *A. fischeri* in the negative controls was strong both before (1a) and after (2b) the field tests. In contrast, the laboratory samples containing fresh 200 ppm DBNPA added to *A. fischeri* cultures (1b) were found to eliminate the bacteria completely. Bioluminescence results from the samples in the fermentation process indicated that the DBNPA efficacy waned. These were measured in duplicate and averaged 95% degradation in trial #1; 98% in trial #2. In each case the results are almost identical to the negative controls, meaning the DBNPA was degraded.

## Discussion

Chemically, two well-characterized degradation pathways have been described for DBNPA: pH-dependent hydrolysis and light-catalyzed reactions with nucleophilies [5]. However, in the case of this study, neither of these mechanisms seem likely. Because the sample was kept in the dark during the degradation study, the light-catalyzed reaction could not occur. The pH-dependent degradation mechanism is rapid at pH > 7; however, the pH of the whole stillage used in this study was 4.6, which indicates that DBNPA is relatively stable. Additionally, the observed degradation rate at this pH was much faster than would be predicted based on the previously reported rate coefficient [11]. Instead, DBNPA may have reacted with nucleophilic compounds in whole stillage, such as sulfhydryl groups derived from corn protein or yeast metabolic byproducts. Physiologically, it is known that DBNPA attacks sulfhydryl groups of bacterial cell proteins, rendering them inactive [4]. The lag between DBNPA disappearance and bromide appearance suggests that one or more intermediate products accumulate during the degradation mechanism followed by a degradation step that releases bromide ions. However, more work needs to be done to define the chemical pathways.

We chose LC/MS/MS for accuracy, precision, and reproducibility and developed a method to measure DBNPA. Based on the analytical chemistry results in Figs. 1 and 2, we conclude that DBNPA decayed rapidly in the whole stillage, which is typical of the fermented material made in the production of bioethanol from corn. The DBNPA applied in the fermentation process was not persistent. The degradation rate of DBNPA in whole stillage was discovered to display first-order kinetics with a calculated half-life of 85 min. During decomposition, an intermediate product forms and, ultimately, decays to free bromide and other (unidentified) compounds by an unknown reaction mechanism. The measured decay rate of DBNPA ignored the effects of sunlight, a major route of rapid decomposition of DBNPA. In addition, whereas in whole stillage at pH (4.6), where DBNPA is expected to be relatively stable and does not undergo rapid degradation through a mechanism of hydrolysis, photolytic and hydrolytic effects may increase the rate of DBNPA decomposition in a manufacturing environment. Consequently, based on the compelling results of the analytical chemistry studies of DBNPA degradation, we pursued further testing in corn-to-ethanol processing plants.

To carry out these field studies, we strove to apply experiences we gained testing the persistence of DBNPA in other field studies. For example, we previously tested the degradation of brominated biocides 2,2-dibromo-3-nitrilo-propionamide and 2-bromo-2-nitro-propane-1,3-diol in cooling water and paper processing industries. These tests were performed by investigating the cellular toxicity of their residuals on *Aliivibrio fischeri* by measuring the change in bioluminescence spectrophotometrically. Likewise, in this study, we conducted field trials on the persistence of DBNPA in corn-to-ethanol processing using the bioluminescence of *A. fischeri* as our sensitive photometric indicator of toxicity, as determined by the Microtox 500 Analyzer [9, 20, 23].

Based on the results in Fig. 3, the effect of DBNPA on *A. fischeri* bioluminescence, we can state that DBNPA was indeed found degraded in whole stillage samples. The field study results measuring loss of DBNPA activity also correspond well with other process water studies conducted similarly. In previous work, when DBNPA as added to fine paper machines, we discovered that 99–100% was actually degraded before the machine effluent was discharged into freshwater (Wiatr and Burns, unpublished data). Subsequently, DBNPA was discovered to degrade in cooling tower water by a different technique; the acute toxicity of DBNPA was less toxic than the literature has stated [27]. Our laboratory and field study results, moreover, agree with these findings. They can help forecast that the application of DBNPA in fermentation of bioethanol can be done without fears that the biocide would carry over into and contaminate DDGS.

Discoveries of the microbiological efficacy of DBNPA [29, 30, 31, 32] against bacteria coupled with the degradation results found early in the corn-to-ethanol process, as provided by the evidence in this paper, can allow this biocide to replace antibiotics in the corn-to-ethanol biofuel industry. That is of significant value. Not only do these results indicate that DBNPA can be used to protect against bacterial infection of the corn-to-ethanol process, saving on costs of raw materials, finished products, and post bacterial infection clean-outs, but they also suggest that use of this biocide can help prevent antibiotic resistance.

Overuse, underdosage, and other misapplications of antibiotics are known to increase antibiotic resistance by bacteria in the field, regardless of the concentration of antibiotics [8, 14, 28]. Antibiotic resistance is also recognized to occur more likely when bacterial biofilms develop, such as those formed on equipment surfaces [1, 7, 18]. Repeated use of antibiotics in animal feed also translates to microorganisms being exposed to sublethal doses of and developing resistance to these drugs. This is a long-term concern, that antibacterial resistance originates in farm animals [19] and continues, particularly when antimicrobials are present at subtherapeutic levels [13] in the food chain.

Moreover, residual antibiotics in meat were previously found to disrupt its fermentation, increase the risk of infection, and make pathogens less susceptible medically to treatment with antibiotics [18]. It appears that antibiotics found at low concentrations at the end of the ethanol process [3] can likewise cause high levels of antimicrobial resistance [6, 33]. The results in this paper indicate that these problems can be avoided. The application of DBNPA instead of an antibiotic to control bacteria in the ethanol process represents a significant advance in the field because DBNPA breaks down prior to the end of the process and thus cannot enter DDGS used for animal foods. This means that the application of DBNPA can circumvent the bacterial antibiotic resistance problem of FDA concern, [12a, 12b] making it a successful alternative to antibiotics. Then agricultural use of DDGS in feed would represent a safer practice because DBNPA would be degraded, would obviate the use of antibiotics that induce antibiotic resistance in bacteria in bovine, swine, and poultry applications in the food chain.

### Electronic supplementary material

11274_2022_3253_MOESM1_ESM.jpeg (JPEG 83 kb)

11274_2022_3253_MOESM2_ESM.jpeg (JPEG 109 kb)

11274_2022_3253_MOESM3_ESM.jpeg (JPEG 81 kb)

11274_2022_3253_MOESM4_ESM.jpeg (JPEG 94 kb)

11274_2022_3253_MOESM5_ESM.jpg (JPEG 92 kb)

## Author checklist

**x** All authors have read the submitted version of the paper AND have approved its submission.

x Please make sure that you **deposit strains and sequence data** in a recognized data bank *(Does not apply to this research paper.)*

x All authors have double checked their affiliation (with precise location), especially if an email address is used that is not provided by the institution.

x All text, including Abstract, tables, references, footnotes and figure legends, is double-spaced printed.

(NOT 1 1/2 spacing) and uses a full-page width (15 cm) on the equivalent of an A4 sheet.

x The title should be informative and clear. Do not use unspecified, nonstandard abbreviations in the title. Avoid ambiguous expressions such as “The effect of…”. Please ensure that the biological origin of the system under investigation is given in the title.

x Key words or phrases (but not abbreviations) are given in **alphabetical order** after the Abstract. Up to 7 can be used.

x Do not submit abstracts divided in sections, containing non-standard abbreviations or citing references.

x Subheadings in all sections should be clearly indicated but are **NOT** numbered.

x Do not combine Results and Discussion.

X Tables and figures are placed after the list of references. (Tables before figures.)

X Legends to figures are grouped together on a separate sheet(s), which precedes the figures themselves. (The Author Checklist is continued on the next page.)

X Position for each table and figure should be indicated in the text by a marginal note or a clear note between paragraphs in the text. *(Done between paragraphs)*

## Claims/declarations

None of the authors have any claims to benefit financially from this research paper; no competing interests. This work on 2,2-dibromo-3-nitrilopropionamide degradation is primarily academic.

C. L. Wiatr, Ph.D.

J.V. Simpson, Ph.D.

## Roles of each co-author

Dr. J. Simpson—carried out the analytical chemistry experiments in the laboratories at SIU-Edwardsville (NCERC) and wrote the original manuscript, including a few references.

Dr. C. Wiatr—was involved in the overall design of the project, wrote the microbiological sections of the article and much of the discussion section, edited the final drafts, and added the majority of the references.

## Why is this work important?

### Quantification and degradation of 2,2-Dibromo-3-Nitrilopropionaminde (DBNPA) in fermentation coproducts

J.V. Simpson, C.L. Wiatr.

The results of analytical and field studies on DBNPA degradation in this paper indicate that this biocide can now be considered an option for applications in the corn-to-ethanol process. Recently published work demonstrates that DBNPA has strong antimicrobial activity in a fermentation matrix and is successful in controlling bacteria that infect and destroy bioethanol products. At the same time, this biocide does not affect the yeast used in making ethanol. However, chemical studies on the degradation of DBNPA in the bioethanol process were not carried out. The results from studies we conducted in the laboratory and in the field provide compelling evidence that DBNPA can break down in whole stillage, meaning that DBNPA is degraded in the ethanol process prior to reaching dry distiller grains (DDGS). This is important because DDGS, made at the end of the ethanol process, is sold as feed for cows, pigs, and poultry. Currently, antibiotics applied in the process are detected in DDGS. The presence of antibiotics in foods can lead to antibiotic resistance in animals and the food chain. Replacing antibiotics with DBNPA would preclude the use of antibiotics in the ethanol process and finally DDGS, obviating antibiotic resistance.
