# Supplementary figures and images for: Quantification and Degradation of 2,2-Dibromo-3-Nitrilopropionamide (DBNPA) in Bioethanol Fermentation Coproducts
Source: World J Microbiol Biotechnol. 2022 Mar 29;38(5):82. doi: 10.1007/s11274-022-03253-0 (PMC8964648; doi:10.1007/s11274-022-03253-0)

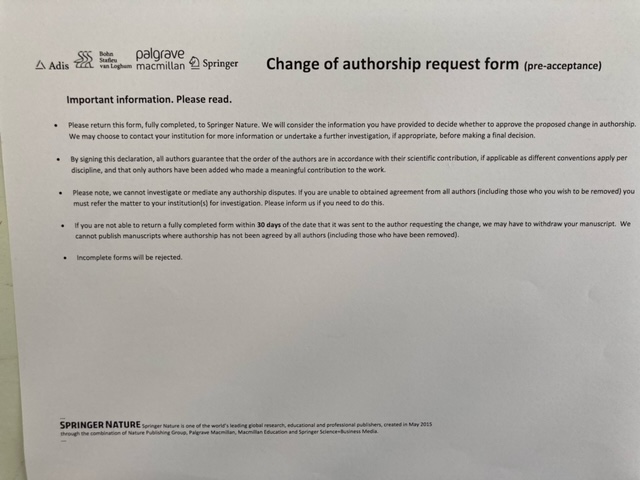

Supplement: Supplementary file 1 — 11274_2022_3253_MOESM1_ESM.jpeg (JPEG 83 kb) [file 11274_2022_3253_MOESM1_ESM.jpeg]

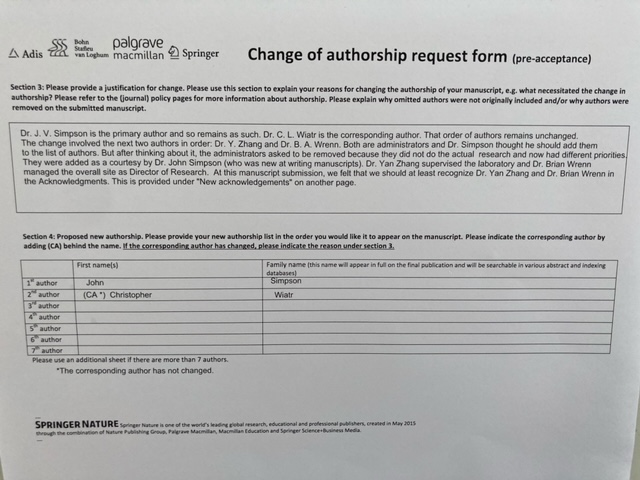

Supplement: Supplementary file 2 — 11274_2022_3253_MOESM2_ESM.jpeg (JPEG 109 kb) [file 11274_2022_3253_MOESM2_ESM.jpeg]

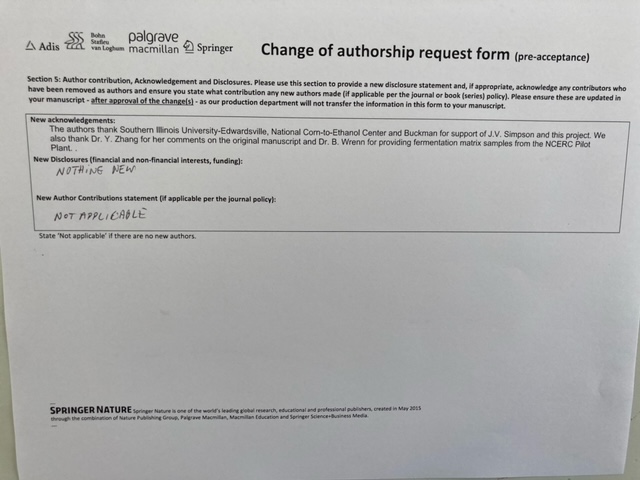

Supplement: Supplementary file 3 — 11274_2022_3253_MOESM3_ESM.jpeg (JPEG 81 kb) [file 11274_2022_3253_MOESM3_ESM.jpeg]

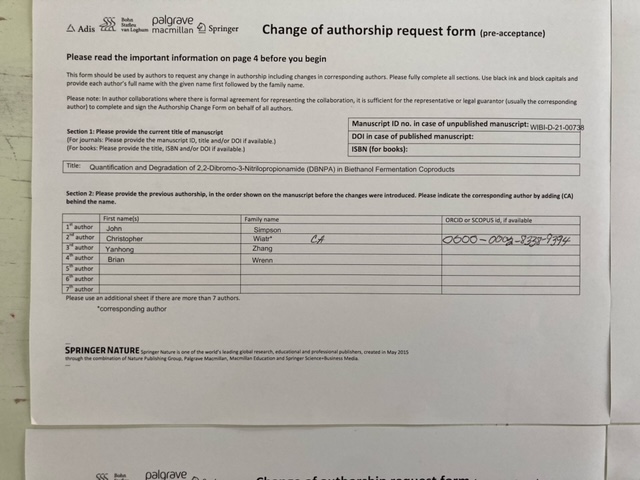

Supplement: Supplementary file 4 — 11274_2022_3253_MOESM4_ESM.jpeg (JPEG 94 kb) [file 11274_2022_3253_MOESM4_ESM.jpeg]

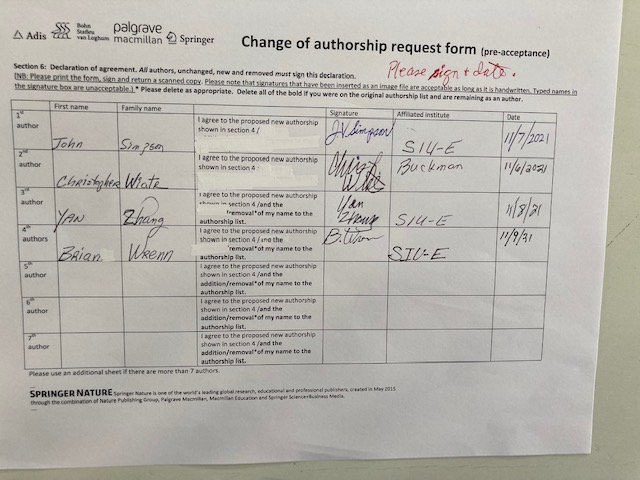

Supplement: Supplementary file 5 — 11274_2022_3253_MOESM5_ESM.jpg (JPEG 92 kb) [file 11274_2022_3253_MOESM5_ESM.jpg]
